# Knowns and unknowns on burden of disease due to chemicals: a systematic review

**DOI:** 10.1186/1476-069X-10-9

**Published:** 2011-01-21

**Authors:** Annette Prüss-Ustün, Carolyn Vickers, Pascal Haefliger, Roberto Bertollini

**Affiliations:** 1 Department of Public Health and Environment, World Health Organization, av. Appia 20, 1211 Geneva 27, Geneva, Switzerland; 2 Regional Office for Europe, World Health Organization, Scherfigsvej 8, DK-2100 Copenhagen Ø, Denmark

## Abstract

**Background:**

Continuous exposure to many chemicals, including through air, water, food, or other media and products results in health impacts which have been well assessed, however little is known about the total disease burden related to chemicals. This is important to know for overall policy actions and priorities. In this article the known burden related to selected chemicals or their mixtures, main data gaps, and the link to public health policy are reviewed.

**Methods:**

A systematic review of the literature for global burden of disease estimates from chemicals was conducted. Global disease due to chemicals was estimated using standard methodology of the Global Burden of Disease.

**Results:**

In total, 4.9 million deaths (8.3% of total) and 86 million Disability-Adjusted Life Years (DALYs) (5.7% of total) were attributable to environmental exposure and management of selected chemicals in 2004. The largest contributors include indoor smoke from solid fuel use, outdoor air pollution and second-hand smoke, with 2.0, 1.2 and 0.6 million deaths annually. These are followed by occupational particulates, chemicals involved in acute poisonings, and pesticides involved in self-poisonings, with 375,000, 240,000 and 186,000 annual deaths, respectively.

**Conclusions:**

The known burden due to chemicals is considerable. This information supports decision-making in programmes having a role to play in reducing human exposure to toxic chemicals. These figures present only a number of chemicals for which data are available, therefore, they are more likely an underestimate of the actual burden. Chemicals with known health effects, such as dioxins, cadmium, mercury or chronic exposure to pesticides could not be included in this article due to incomplete data and information. Effective public health interventions are known to manage chemicals and limit their public health impacts and should be implemented at national and international levels.

## Background

Chemicals are part of our daily lives. On the other hand, they may cause diseases. Which fraction of the current disease burden do chemicals however cause, and which are the chemicals of greatest concern? This is an important question for decision-makers in order to prioritize efforts to protect us from the harmful effects of chemicals.

This article describes and summarizes the main estimates available to date of the health impact of chemicals on the population at global level. It provides their sum and the relative importance of the groups of chemicals contributing to these health impacts. This information on the collective role of chemicals as contributors to global disease may assist policy makers in setting priorities in view of health protection. As the population health impacts from many chemicals have not yet been assessed, an overview of exposures and health impacts is provided to map estimated disease burden to the actual burden and identify data gaps. We also outline the main exposures involved and highlight areas relevant to prevention of exposure.

This review has focused on toxic exposures to chemicals which can be significantly reduced or eliminated through environmental and occupational management. These environmental exposures to chemicals cause a disease burden of unknown magnitude, and have yet been attracting considerable attention of the public, policy makers and research communities. This situation has been the main motivation for undertaking this review, and for defining its focus. Chemical exposures that are not primarily linked to environmental management, as for example active smoking, are not addressed in this review. Radioactive substances have equally not been included here, as they have a different mechanism of action, often concern other interest groups and require a specific set of safety measures. Indirect consequences of chemicals, acting for example through climate change, have not been taken into account.

### Human exposure to chemicals

Chemicals, whether of natural origin or produced by human activities, are part of our environment. Naturally occurring chemicals include arsenic and fluoride in drinking water, suspended particulate matter and sulfur dioxide from volcanic emission or forest fires, or naturally occurring toxins. Manufactured chemicals include industrial and agricultural products such as pesticides, petroleum products, processed metals, and products of combustion such as toxic gases and particles from industrial emissions and burning of fuel. Some chemicals are manufactured for specific uses, while others are unwanted by-products, wastes, or products of combustion.

**Figure 1:**
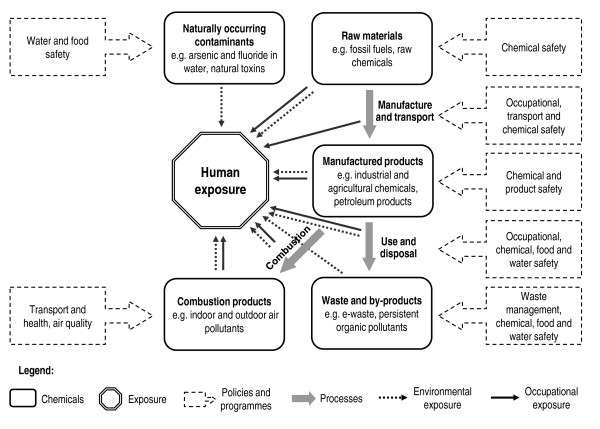
Human exposure to chemicals throughout their life-cycle and selected programmes relevant to their prevention

Their chemical, physical and toxicological properties vary greatly - while many are not hazardous or persistent, some are life-threatening on contact and some persist in the environment, accumulate in the food chain, travel large distances from where they are released, and are harmful to human health in small amounts. Human exposure can occur at different stages of the life-cycle of a chemical, including through occupational exposure during manufacture, use and disposal, consumer exposure, exposure to contaminated products, or environmental exposure to toxic waste (Figure 1). Exposure can occur via various pathways, including inhalation of contaminated air and dust, ingestion of contaminated water and food, dermal exposure to chemical or contaminated products, or fetal exposure during pregnancy (Table 1). Further information on human exposure to chemicals is available from a variety of documents [1-4]. As illustrated in Figure 1, several sectors and programmes have a role to play in preventing human exposure to chemicals and promoting their sound management throughout their life cycle, including health, environment, agriculture, energy and transport sectors, and water, food and chemical safety programmes.

**Table 1:** Examples of sources and pathways of human exposure to a few selected chemicals

Exposure media	Example sources of exposure and exposure pathways	Examples of chemicals
Outdoor air	Inhalation of toxic gases and particles from vehicle and industrial emissions, or naturally occurring sources such as volcanic emission or forest fires.	Sulfur dioxide, nitrogen oxides, ozone, suspended particulate matter, lead, benzene, dioxins and dioxins-like compounds

Indoor air	Inhalation of pollutants released during indoor combustion of solid fuels, tobacco smoking, or from construction materials and furnishings, contaminants in indoor air and dust.	Suspended particulate matter, nitrous oxide, sulfur oxides, carbon monoxide, formaldehyde, polyaromatic hydrocarbons (PAH), mercury, lead dust from lead-based paints, benzene, asbestos, mycotoxins, phtalates, polybrominated diphenyl ether fire retardants (PBDEs)

Drinking water	Ingestion of drinking water contaminated with toxic chemicals from industrial effluents, human dwellings, agricultural runoff, oil and mining wastes, or from natural sources.	Pesticides, herbicides, fertilisers, metals (copper, lead, mercury, selenium, chromium), arsenic, fluoride, nitrate, cyanide, industrial solvents, petroleum products, disinfection by-products.

Food	Consumption of food contaminated with chemicals at toxic levels through agricultural practices, industrial processes, environmental contamination, and natural toxins.	Pesticides, methylmercury, lead, cadmium, dioxins, aflatoxin.

Non-food consumer products	Exposure by ingestion, inhalation or dermal exposure to toxic chemicals contained in toys, jewellery and decoration items, textiles, or food containers, consumer chemical products	Lead, mercury, cadmium, phthalates, formaldehyde, dyes, fungicides or pesticides.

Soil	Ingestion (particularly for children) or inhalation of soil contaminated through industrial processes, agricultural processes or inadequate household and industrial waste management.	heavy metals, pesticides, and persistent organic pollutants.

Occupational exposure	Chronic or acute exposures through inhalation, dermal absorption, or secondary ingestion of toxic chemicals or by-products of industrial processes such as agriculture, mining or manufacturing.	Pesticides, benzene, heavy metals, solvents, suspended particulate matter.

Human to human	Foetal exposure to toxic chemicals during pregnancy (through placental barrier) or through consumption of contaminated breast milk.	Heavy metals, pesticides, benzene, etc.

## Methods

We systematically reviewed the published literature for studies estimating the disease burden, expressed in deaths or DALYs (a measure combining mortality and morbidity), either at global level or covering a large part of the total burden (i.e. national level estimates were generally not retained). We reviewed the disease burden estimates developed by the World Health Organization and those published in the peer reviewed literature. We used combinations of the terms of main chemicals of concern or their mixtures (which might be expected to have broad exposure information and epidemiological evidence, see Additional file 1: Search terms used in Pubmed), "global", "world health", "burden", "impacts", "effects" and "review" in PubMed Databases and Google from 1990 to December 2009.

Relevant estimates were found for 14 chemicals or their groups or mixtures. Most estimates were developed by groups of experts coordinated by the World Health Organization. Methods underlying the estimates were reviewed and summarized. For estimating the total known burden of disease from chemicals, methods were reviewed for compatibility, summed up and categorized into broad exposure groups. Estimates from overlapping exposures (i.e. compounds appearing again in mixtures) were removed.

In order to map the available estimates of disease burden to the total burden of disease from chemicals we reviewed the literature to develop a basic frame. Table 2 lists the main health outcomes associated with exposure to toxic chemicals to illustrate this wide range of diseases.

**Table 2:** Main disease groups with suspected or confirmed linkage to chemicals

Diseases/disease groups	Examples of exposures	**Examples of associated outcomes **[22,66,67]
Respiratory infections and chronic respiratory diseases	Occupational exposures to dusts, gases, irritant chemicals, fumes	Chronic Obstructive Pulmonary Disease (COPD) [68,69]
	Second-hand smoke; occupational exposures to cleaning-agents, pesticides, hairdressing chemicals etc.	Asthma onset and exacerbation [28,70-72]
	Second-hand smoke	Acute lower respiratory infections [28]
	Occupational exposure to asbestos Metal dusts, particulate matter	Asbestosis Bronchitis, pneumoconiosis, silicosis

Perinatal conditions	Maternal exposure to pesticides or other chemicals	Low-birth-weight and preterm infants [73-76]

Congenital anomalies	Maternal exposure to pesticides, polychlorinated biphenyls (PCBs), polychlorinated dibenzofurans (PCDFs), lead, mercury, other endocrine disruptors	Various birth defects [77,78]

Diseases of the blood	Lead, arsine, naphthalene, benzene	Anaemia, methaemoglobinemia

Cancers	Occupational exposures to carcinogens, aflatoxins in food, second-hand smoke, outdoor air pollution by carbon particles associated with polycyclic aromatic hydrocarbons, asbestos, arsenic; volatile organic compounds such as benzene, pesticides, dioxins. etc.	Numerous cancer sites, including of the lung, skin, liver, brain, kidney, prostate, bone marrow, bladder [79-82]

Neuropsychiatric and developmental disorders	Lead, methylmercury, polychlorinated biphenyls (PCBs), arsenic, toluene etc.	Cognitive development, mental retardation, Parkinson disease, Attention-deficit disorder, Minamata disease [51,78,83,84]

Sense organ diseases	Carbon disulfide, mercury, lead	Hearing loss

Cardiovascular diseases	Ultrafine particles in polluted air, lead, arsenic, cadmium, mercury, pollutant gases, solvents, pesticides, second-hand smoke	Ischaemic heart disease, cerebrovascular disease [28,49,85]

Diabetes mellitus	Arsenic, N-3-pyridylmethyl-N'-p-nitrophenyl urea (rodenticide), 2,3,7,8-Tetrachlorodibenzo-p-dioxin.	Diabetes Type II [86-89]

Systemic auto immune diseases	Crystaline silica dust	Systemic sclerosis, systemic lupus erythematosus, rheumatoid arthritis, systemic small vessel vasculitis [90]

Endocrine diseases	Ethanol, hexachlorobenzene	Porphyria

Genito-urinary diseases	Beryllium, cadmium, lead	Calculus of kidney, chronic renal disease

Digestive diseases	Ethanol, chloroform, carbon tetrachloride, manganese	Hepatitis, cholestasis, pancreatitis

Skin diseases	Antiseptics, aromatic amines, cement, dyes, formaldehyde, artificial fertilizers, cutting oils, fragrances, glues, lanolins, latex, metals, pesticides, potassium dichromate, preservatives	Atopic dermatitis, allergic and irritant contact dermatitis, chloracne, hyperkeratosis [91]

Musculoskeletal diseases	Cadmium, lead	Osteoporosis, gout

Oral conditions	Fluoride	Dental fluorosis

Poisonings	Accidental ingestion of household products, occupational exposures and accidents, intentional self-harm by ingestion of pesticides	Unintentional poisonings, self-inflicted injuries [92-94]

### Methods underlying the burden of disease estimates

In most disease burden estimates for risk factors, the basic method can be summarized in the following steps: (a) estimating the exposure distribution in a population; (b) selecting one or more appropriate relative risk estimates from the literature, generally from a recent meta-analysis; and (c) estimating the population attributable fraction (simplified formula in Panel 1 [5,6]). The resulting population attributable fractions, estimated for each disease, age group, gender, and population group, are then multiplied by the total number of deaths and disability-adjusted life years (DALYs) for the disease in each population group. DALYs are a weighted measure of deaths and disability [7].

Panel 1: Population attributable fraction

$$PAF=\frac{p(RR-1)}{p(RR-1)+1}$$

RR = relative risk; p = prevalence of exposure

The available disease burden estimates have been developed with variations of the above method, or different approaches supported by differing levels of evidence. Additional features on the various approaches used are detailed as follows:

#### Comparative Risk Assessment (CRA)

The method systematically assesses changes in population health by varying distribution of exposure using a unified framework linked to the Global Burden of Disease [7]; Developed by WHO and numerous experts, [8] this study included risk factors causing more than 0.5% of the global disease burden, with high likelihood of causality, and reasonably complete exposure data.

CRA approaches involve estimating the disease burden compared to a "counterfactual" exposure distribution which will generally lead to the lowest conceivable disease burden, irrespective of whether currently attainable in practice [9]. For example, the counterfactual exposure for outdoor air pollution was the natural background level of particulate matter, and zero for second-hand smoke. The disease burden estimates essentially represent the burden that could be prevented if the risk factors were removed. Additional details of the methods are provided in greater detail in the original publications [8-10].

Limitations of the CRA approach include (a) the limited scope by addressing only risk factors with large impacts at global level; (b) the restriction to high level of evidence, not taking account of "likely" impacts (which can also be seen as an advantage) and therefore generally underestimating the total impacts; (c) the inclusion of only those diseases defined through the International Classification of Diseases (ICD), rather than all health effects. For example loss of IQ points from lead (unless resulting in mental retardation) has not been taken into account, as it is not a disease according to ICD.

#### Other exposure-based estimates than CRA

Some estimates use methods similar to the CRA but not necessarily a unified framework [11,12]. This means that the choice of outcomes and exposure-risk relationships does not necessarily underlie specific selection criteria, and that different approaches are used in terms of counterfactual scenarios (i.e. alternative exposure scenario). Such assessments may be equally rigorous as compared to the CRA, but results may not be comparable or additive with CRA estimates.

#### Estimates based on evidence synthesis completed with expert opinion

These methods combine CRA estimates and evidence synthesis from partial or geographically limited assessments, and fill knowledge gaps with expert estimates. This approach may provide approximate estimates when global exposure information is limited, or when quantitative exposure-risk relationships are supported by weaker evidence. Rigorous estimates can so be completed to obtain a fuller picture of likely population health impacts from various risks. The main disadvantage obviously consists in the lower level of evidence supporting those estimates. Further details on the method are provided in the original publication [13].

Before adding up the estimated burden of disease from various chemicals, any joint effects of different risks need to be considered. The burden of disease estimates describe the burden that would be removed if exposure was reduced to the counterfactual exposure [8]. As diseases may be caused by multiple factors, the estimated disease burdens could add up to more than 100%. For example, one childhood deaths of respiratory infection of an underweight child could be prevented both by removing the source of indoor air pollution, or by improved nutrition [8]. Deaths from those two risk factors should therefore not be added up. In the case of the chemicals identified in this article, however, significant overestimation of the burden due to joint effects is unlikely: few exposures are expected to overlap significantly and at the same time cause synergistic effects or health impacts that could be avoided by reducing either of those exposures. For greater consistency with previous estimates and better comparability and additivity, we preferred CRA types of assessments and others using a counterfactual exposure.

## Results

The systematic literature review revealed burden of disease estimates for the following chemicals or groups of chemicals: (a) chemicals involved in unintentional acute poisonings, (b) chemicals involved in unintentional occupational poisonings, (c) pesticides involved in self-inflicted injuries, (d) asbestos, (e) occupational lung carcinogens, (f) occupational leukaemogens, (g) occupational particulates, (h) outdoor air pollutants, (i) indoor air pollutants from solid fuel combustion, (j) second-hand smoke, (k) lead, and (l) arsenic in drinking water. The following paragraphs describe and discuss available estimates for these chemicals. An overview of the global burden of disease attributable to these chemicals is presented in Table 3. More detailed descriptions of the methods used for the presented burden of disease estimates are found in the original referenced publications. Most of the identified burden of disease estimates from chemicals follow CRA methods and provide data for the year 2004 [14], which was used as the reference year in this article.

**Table 3:** Overview of available disease burden estimates attributable to chemicals

Chemicals/Groups of chemicals	Disease outcomes considered (attributable fraction)	Deaths	DALYs^‡^	Main limitations¤	Data year/method^§^
*Chemicals in acute poisonings*		*526,000 (sub-total)*	*9,666,000 (sub-total)*		

Chemicals (including drugs) involved in unintentional acute poisonings (methanol, diethylene glycol, kerosene, pesticides etc.)	Unintentional poisonings (71%)	240,000^a^	5,246,000^a^	Limited to preventable poisonings. Total unintentional poisonings would amount to 346,000 deaths and 7,445,000 DALYs[12]	2004; C [13]^b^

Chemicals involved in unintentional occupational poisonings	Unintentional poisonings (occupational) (8.6%)	30,000^c^	643,000^c^	-	2004; A [14]

Pesticides pesticides involved in self-inflicted injuries	Self-inflicted injuries (23%)	186,000	4,420,000	Limited to preventable self inflicted injuries. Impact of accidental and chronic exposures not considered.	2002; C [13]

*Chemicals in occupational exposures (longer term effects)*		*581,000 (sub-total)*	*6,763,000 (sub-total)*		

Asbestos	Malignant mesothelioma (NA); trachea, bronchus, lung cancer (0.3%); asbestosis (NA)	107,000^d^	1,523,000^d^	-	2004; A [14]^, ^[50]

Occupational lung carcinogens (arsenic, asbestos, beryllium, cadmium, chromium, diesel exhaust, nickel, silica)	Trachea, bronchus, lung cancer (8.6%)	111,000	1,011,000	Only 8 of the chemicals or chemical mixtures classified as carcinogenic or probably carcinogenic to humans taken into account	2004; A [14]

Occupational leukaemogens (benzene, ethylene oxide, ionizing radiation)	Leukaemia (2.3%)	7,400^e^	113,000^e^	Only 2 of the chemicals or chemical mixtures classified as carcinogenic or probably carcinogenic to humans taken into account	2004; A [14]

Occupational particulates - causing COPD (dusts, fumes/gas)	COPD (13%)	375,000^f^	3,804,000^f^	-	2004; A [14]

Occupational particulates - other respiratory diseases than COPD (silica, asbestos and coal mine dust)	Asbestosis (NA); silicosis (NA); pneumoconiosis (NA)	29,000	1,062,000	-	2004; A [14]

*Air pollutant mixtures*		*3,720,000 (sub-total)*	*60,669,000 (sub-total)*		

Outdoor air pollutants (particulate matter, sulfur dioxide, nitrogen oxides, benzo[a]pyrene, benzene, others)	Lung cancer (7.9%); acute respiratory infections (1.6%); selected cardiopulmonary diseases (3.4%)	1,152,000	8,747,000	Only urban air pollution in cities with >100 000 inhabitants taken into account. Health impact from rural air pollution unknown.	2004; A [14]

Outdoor air pollutants emitted from ships (particulate matter, sulfur dioxide, nitrogen oxides, benzo[a]pyrene, benzene, others)	Lung cancer (0.3%); selected cardiopulmonary diseases (0.4%)	60,000^g^	NA	-	2002; B [95]

Indoor air pollutants from solid fuel combustion (carbon monoxide, nitrogen oxides, sulfur oxides, benzene, formaldehyde, polyaromatic compounds, particulates, others)	Lung cancer (2.9%); acute respiratory infections (33%); COPD (33%)	1,965,000	41,009,000	Disease burden from emissions from building materials and household products is not know. BoD from second hand smoke has been evaluated separately.	2004; A [14]

Second-hand smoke (nicotine, formaldehyde, carbon monoxide, phenols, nitrogen oxides, naphthalenes, tar, nitrosamine, PAHs, vinyl chloride, various metals, hydrogen cyanide, ammonia, others)	Lower respiratory infections (6.3%); otitis (1.7%); asthma (11%); lung cancer (1.8%); ischaemic heart disease (4.5%)	603,000	10,913,000	-	2004; B [29]

*Single chemicals with mostly longer term effects*		*152,000 (sub-total)*	*9,102,000 (sub-total)*		

Lead	Mild mental retardation; Cardiovascular diseases	143,000	8,977,000	-	2004; A [14]

Arsenic in drinking-water	Diabetes mellitus (0.04%) ischemic heart disease (0,11%); lung cancer (0.25%); bladder cancer (1.2%); kidney cancer (NA); skin cancer (0.30%)	9,100^a^	125,000^a^	Limited to exposure through drinking water. Limited to Bangladesh.	2001; B [11]

Total ^#,h ^Total in children <15 years	All considered diseases	4,879,000 (8.3%) 1,073,000 (22%)	86,200,000 (5.7%) 46,627,000 (54%)		Mainly 2004; A

^‡ ^DALYs are "Disability-adjusted life years", a weighted measure of years of life lost due to premature death, and years lived with disability. ¤ Only outcomes qualified as strong evidence were considered. ^§ ^Methods: A: Comparative Risk Assessment (CRA); B: Based on exposure and exposure-response (similar to CRA); C: Evidence synthesis and expert evaluation. ^# ^The estimates were developed within three years and their pooling is unlikely to introduce a significant error. NA: not available. -: none.

^a ^Estimate not compared to counterfactual exposure, which is however estimated to be negligible using a theoretical minimum exposure given available management options for concerned chemicals.

^b ^Values updated for 2004 based on original reference.

^c ^Already included in total unintentional acute poisonings and therefore not included again in the total.

^d ^Lung cancer and asbestosis caused by asbestos are also considered in occupational lung carcinogens and particulates and this part of the burden is therefore not counted twice in the total.

^e ^Also includes a small fraction of leukaemia caused by ionizing radiation.

^f ^Parts of the particulates are organic in nature, and the estimate therefore includes a small fraction that is not or not directly related to chemicals

^g ^Overlaps with the burden from outdoor air pollution and is therefore not included in the total.

^h ^Total is corrected for double counting (chemicals considered in more than one estimate); not all disease burdens are however additive, and joint exposures could lead to slight overestimate (see Methods section).

### Chemicals in unintentional acute poisonings

Unintentional ingestion, inhalation or contact with chemicals caused 346,000 deaths (7,447,000 DALYs) from acute poisonings in 2004. About 71% of unintentional poisonings were estimated to be preventable through improved chemical safety [13], amounting to 240,000 deaths and 5,246,000 DALYs in 2004. The share of this disease burden affecting children amounts to 19%, and 30,000 deaths were estimated to occur at the workplace. Chemicals responsible for unintentional poisonings may include methanol, diethylene glycol, kerosene, pesticides, and many others. Original methods for occupational poisonings were developed in the CRA framework [15]. This estimate also includes inadequate use of pharmaceuticals, which is however likely to be a minor contributor.

### Pesticides in self-inflicted injuries

A large body of evidence supports the causation of various diseases (e.g. cancers, birth defects) and other health effects (e.g. endocrine disruption, neurotoxicity, kidney/liver damage) by exposure to pesticides [16]. Both exposure and exposure-response data available are unfortunately too limited to estimate the global health impacts of pesticides. The global impact of self-poisoning (suicides attempts) from preventable pesticide ingestion was, however, estimated to amount to 186,000 deaths and 4,420,000 DALYs in 2002 (analysis of evidence complemented by expert opinion [13]). The total burden of suicides from pesticide ingestion was estimated to amount to 258,000 deaths in 2002 with a more rigorous approach, but the former estimate is used in this context for methodological reasons (includes a counterfactual approach, to allow for additivity of results) [12]. Current evidence supports that suicide rates could be significantly reduced through limiting access to lethal means, among other methods [17,18]. About 30% of self-inflicted injuries globally involve pesticides, and occur mainly in Asia and to a lesser extent in almost all other parts of the world.

### Asbestos

Exposure to asbestos causes lung cancer, mesothelioma and asbestosis (fibrosis of the lungs), and other outcomes [19]. The global burden of disease attributable to asbestos has been estimated to amount to 107,000 deaths and 1,523,000 DALYs for the three mentioned diseases in 2004. Among these, 41,000 deaths and 370,000 DALYs were due to asbestos-caused lung cancer, and 7,000 deaths and 380,000 DALYs to asbestosis. The remaining 59,000 deaths and 773,000 DALYs were attributed to malignant mesothelioma [14,15,20]. Deaths and DALYs from lung cancer and asbestosis are also included in the estimates for occupational lung carcinogens and occupational particulates, and are therefore not counted twice when summing the total disease burden from chemicals in Table 3.

### Occupational lung carcinogens

Occupational exposure to arsenic, asbestos, beryllium, cadmium, chromium, diesel exhaust, nickel and silica were estimated to cause 111,000 deaths (and 1,011,000 DALYs) from lung cancer in 2004 [14]. This represents about 9% of the total burden of lung cancer. Health impacts from additional lung carcinogens, such as bis(chloromethyl)ether, 2,3,7,8-TCDD, soot exposure while chimney sweeping, aluminium production, iron and steel founding, rubber manufacturing among others could not be estimated [21].

### Occupational leukaemogens

Benzene, ethylene oxide and ionizing radiation were the only occupational leukaemogens considered in the estimation of global burden of disease from occupational leukaemogens [15]. This analysis resulted in a total of 7,400 deaths (and 113,000 DALYs) in 2004 [14], representing 2.3% of the total burden of leukaemia. IARC has classified few additional chemicals or exposures as supported by sufficient evidence in their association with leukaemia, including formaldehyde and exposures in the rubber manufacturing industry [21].

### Occupational particulates

Exposure to particulate matter has been linked to a vast range of respiratory and other diseases. The estimation of disease burden from occupational exposure to particulate matter was limited to selected respiratory diseases as exposure and risk information at global level is limited [15]. Effects from exposure to dust and/or gas/fumes on COPD, and from exposure to silica, asbestos and coal mine dust on silicosis, asbestosis and coal workers' pneumoconiosis were considered. The estimated health impacts were 375,000 deaths and 3,804,000 DALYs from COPD, and 29,000 deaths and 1,061,000 DALYs from asbestosis, silicosis and pneumoconiosis in 2004 [14,20]. Occupational agents associated with the development of COPD include for example mineral fumes, welding fumes, cadmium fumes and sulfur dioxide [22]. The fraction of COPD attributable to occupational particulates is estimated to amount to 13% globally. Some dusts of biological nature, such as cotton, grain and wood dusts, are also suspected to have a role in COPD causation [22]. Despite the possible inclusion of biological dusts, this estimate may still be an underestimate as only part of the respiratory diseases caused by chemicals were considered.

### Outdoor air pollutants

Outdoor air pollution contains particulate matter and gaseous pollutants, such as sulfur dioxide (SO_2_), nitrogen oxides (NO_x_) and carbon monoxide (CO), as well as secondary pollutants such as ozone (O_3_) formed from directly emitted pollutants. Further constituents of the pollutant mixture include carcinogens such as benzo[*a*]pyrene, benzene and 1,3-butadiene [23]. Health impacts from urban air pollution, largely from combustion sources, caused overall about 1,152,000 deaths (8,747,000 DALYs) worldwide in 2004 [14,24]. In particular, respiratory infections in children contributed 121,000 deaths (1,555,000 DALYs) to this burden, lung cancer 108,000 deaths (931,000 DALYs) and other cardiopulmonary illnesses 923,000 deaths (6,261,000 DALYs). About 10% of this burden is estimated to affect children. Exposure was measured, and modeled when not available, using particulate matter (PM_10 _and PM_2.5_) as an index for common mixtures of urban air pollution [24]. These estimates cover cities with >100,000 inhabitants. Rural outdoor air pollution, e.g. caused by forest fires [25] or indoor combustion of solid fuels, may also contribute to global health impacts and have not been estimated.

### Indoor air pollutants from solid fuel combustion

Indoor air pollution is caused by both traditional sources of pollution, primarily by the combustion of solid fuels for cooking or heating, and modern sources such as building materials and household products emitting chemicals. Household combustion of coal or biomass produces smoke that contains carbon monoxide (CO), nitrogen oxides (NO_x_), sulfur oxides (SO_x_), benzene, formaldehyde, polyaromatic compounds, and particulates and many more [26]. Building materials and household products can release toxic chemicals such as benzene and formaldehyde. About half of the world's households still use solid fuels, which were estimated to cause 872,000 deaths (30,854,000 DALYs) from lower respiratory infections in children, 36,000 deaths (338,000 DALYs) from lung cancer, and 1,057,000 deaths (9,817,000 DALYs) from COPD in 2004 [14]. With 75%, children bear the greatest burden from exposure to these pollutants. This estimate only covered indoor smoke from solid fuel combustion, and did not address emissions from building materials and household products, such as benzene and formaldehyde, and other indoor air contaminants.

### Second-hand smoke

Second-hand smoke is a complex mixture of compounds emanating from tobacco smoke causing indoor air pollution. These include more than 30 known or suspected human carcinogens, such as 4-aminobiphenyl, 2-aminonaphthalene, benzene, nickel, and a variety of polycyclic aromatic hydrocarbons (PAHs) and *N*-nitrosamines [27]. A number of irritants, such as ammonia, nitrogen oxides, sulfur dioxide and various aldehydes, and cardiovascular toxicants, such as carbon monoxide, nicotine and some PAHs, are also present [28]. Second-hand smoke was estimated to cause 166,000 deaths (6,616,000 DALYs) in children in 2004, and 436,000 deaths (4,297,000 DALYs) in non-smoking adults [29], which means that 61% of the burden is borne by children.

### Lead

Human exposure to lead contributes mainly to cardiovascular diseases, mild mental retardation from childhood exposure leading to reduced intellectual function, and additional outcomes which are more difficult to quantify [30]. The 2004 global burden of disease for these outcomes was estimated to be 143,000 deaths and 8,977,000 DALYs [14]. Among those, all deaths and 1,789,000 DALYs were due to lead-induced cardiovascular diseases in adults, and 7,189,000 DALYs were a result of mild mental retardation due to lead-associated IQ deficits, which means that children carried 80% of the disease burden from lead. Childhood lead exposure was estimated to contribute to about 600,000 new cases of children with intellectual disabilities every year. Between 2000 [31] and 2004, the proportion of the global population with blood lead levels above 10 ug/dl decreased from 20% to 14%, and resulted in similar reductions in the disease burden, mainly due to important efforts in phasing out leaded gasoline in most countries. It should be noted that blood lead levels can cause disease well below 10 ug/dl. Other significant sources of lead exposure however persist and continue to contribute significantly to the overall disease burden [32,33]. Additional confirmed or suspected outcomes (see Table 2) or health impacts from "hot spots" (e.g. locally elevated exposures from industrial activities) were not included in the estimate.

### Arsenic in drinking-water

Human exposure to arsenic can cause a variety of health effects and diseases, including cancer of the skin, bladder, kidney and lungs. Other effects of long-term exposure are peripheral neuropathy, gastrointestinal symptoms, diabetes, reproductive effects, enlarged liver, bone marrow depression, destruction of erythrocytes, high blood pressure and cardiovascular disease [34-36]. Exposure to arsenic can occur through different environmental pathways, including from mining and smelting activities, burning of arsenic-rich coal and ingestion of contaminated drinking-water. Arsenic mainly enters drinking-water supplies through natural deposits in the soil, but also through industrial and agricultural activities (including discharge of industrial wastes, burning of fossil fuels - especially coal - and wastes, or use of pesticides and food additives). The health impacts of exposure to arsenic in drinking water have been estimated for Bangladesh [11]. Another estimate has been performed at global level, but is not comparable [37]. Arsenic-contaminated drinking-water in Bangladesh alone contributed 9,100 deaths and 125,000 DALYs in 2001 from diabetes mellitus, ischaemic heart disease, lung cancer, and bladder, kidney and skin cancer [11]. About two thirds of the total population exposed to elevated drinking-water levels are estimated to reside in Bangladesh [11]. The estimated burden of disease therefore represents a significant part of the global burden from arsenic in drinking water. A global estimate, or an estimate for other exposures than drinking water was not available for arsenic.

#### Other health impact estimates available

Additional assessments of global health impacts of selected chemicals have been made but results are not comparable to the other analyses compiled here, either because they were not expressed in DALYs and deaths, or because DALYs have not been estimated in a comparable format. Examples include fluoride and mercury. They are presented in the following paragraphs.

### Fluoride

Insufficient fluoride intake increases the risk to develop dental caries, while excessive intake can lead to dental and skeletal fluorosis. High concentrations of fluoride can enter public water systems from natural sources, including runoff from the weathering of fluoride-containing rocks and soils and leaching from soil into groundwater. Fluoride pollution from various industrial emissions can also contaminate water supplies [38]. Excessive fluoride concentrations in drinking water was estimated to have caused about 47 million of dental fluorosis cases and 20 million skeletal fluorosis cases in 17 countries (in terms of prevalence, based on point estimates published between 1953 and 2000) [39].

### Mercury

Mercury compounds are toxic to the nervous, digestive, cardiovascular and immune systems, to the lungs, kidneys, skin, eyes, and gastrointestinal tract, and adversely impact on development [40,41]. Mercury releases in the environment result mainly from human activity, for example emissions from coal-fired power plant, and the use of mercury-containing products. Once in the environment, elemental mercury is naturally transformed into methylmercury that bioaccumulates in fish and shellfish. Human exposure occurs mainly through inhalation of elemental mercury vapors during industrial processes and through consumption of contaminated fish and shellfish. Transplacental exposure of the foetus may also occur [42]. The analysis of disease burden from methylmercury was limited to cognitive impacts and mild mental retardation. It was estimated that, among selected subsistence fishing populations, between 1.5/1000 and 17/1000 children showed cognitive impacts caused by the consumption of fish containing methylmercury. These results are however not comparable to estimates of deaths and DALYs [41,43].

#### Results on data used in the burden of disease estimates

Exposures used in the burden of disease estimates have included (a) international databases or systematic reviews of monitoring/modelling of environmental levels, such as for outdoor air pollution [44,45] and arsenic [11,37] (b) international databases or systematic reviews of survey data, such as for indoor air pollution [46] and second-hand smoke [29] (c) systematic reviews of bio-monitoring data, such as blood lead levels [31,47], or (d) international databases or systematic reviews of occupational exposures by sector or occupational category, such as for occupational carcinogens [48] or occupational particulates [15]. These exposure data have been combined with exposure-risk information from major reviews or epidemiological studies to result in burden of disease estimates. Examples include lead and hypertension or cardiovascular effects [49,50]; lead and children's intellectual function [51,52]; outdoor air pollution and cardiopulmonary disease [53]; second-hand smoke and related outcomes [27,28,54]; arsenic in drinking-water and related outcomes [55]; occupational exposure and lung cancer [56] or asbestos [57]). The estimation of acute effects from chemicals at population level has generally been based on the direct assessment of cases or deaths, such as mortality statistics for unintentional poisonings [58] and systematic reviews of vital statistics, autopsy reports, surveillance systems, hospital-based studies etc. for suicides involving pesticides [12].

## Discussion

### Estimated burden of disease from chemicals

**Figure 2:**
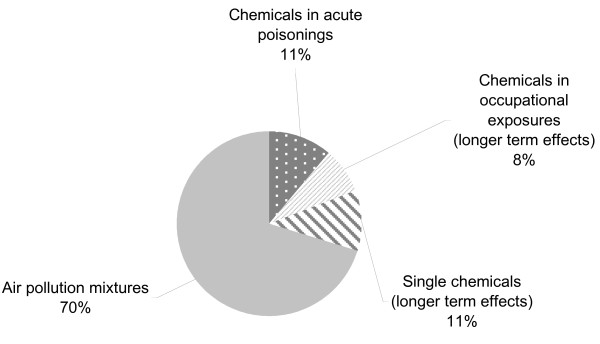
Distribution of known burden of disease (in DALYs)

This review shows that, based on estimations available to date, the global burden of disease attributable to environmental exposure and management of selected chemicals amounts to at least 4.9 million deaths (86 million DALYs) per year. This represents 8.3% of the total deaths and 5.7% of the total burden of disease in DALYs worldwide. For comparison, this is more than the burden of all cancers worldwide, which account for 5.1% of all DALYs [58]. Fifty-four percent of this burden (counted in DALYs) is borne by children under the age of 15 years. The share of the total disease burden is considerable, and supports the need for further public health considerations in this area. By far the largest disease burden is related to exposure to air pollution mixtures with 70% of the total (Figure 2). Our estimate includes available information for chemicals in a broad sense, i.e. not only industrial and agricultural chemicals but also air pollutants and some naturally occurring chemicals. Available information for industrial and agricultural chemicals and acute poisonings only (i.e. without air pollution nor arsenic-contaminated drinking-water) amounts to a global burden of disease of at least 1.2 million deaths (25 million DALYs), corresponding to 2.0% of the total deaths and 1.7% of the total burden of disease worldwide.

### Limitations

The global estimates presented in this article undoubtedly underestimate the real burden attributable to chemicals. Comparing the identified disease burden from chemicals with the health effects listed in Table 2 shows that only limited relevant exposures and some of the health impacts they cause have so far been quantified at population level. There are several reasons for this underestimate: (a) Quantified exposure-response relationships, supported by strong evidence, between many chemicals and their health outcomes are lacking. Establishing further links between certain chemicals and their health hazards would be useful but may be complex; (b) Large-scale exposure data are insufficient. Even chemicals with health consequences supported by strong evidence of causality therefore bear considerable knowledge gaps in terms of population impact; (c) This analysis also failed to capture much of the health impacts from exposure to polluted sites which are estimated to put at risk more than 56 million people worldwide [59]; such locally-specific health impacts are difficult to estimate with available methods and should be considered separately.

As this review is mostly built on previous estimates developed by WHO - given the limited availability of other estimates - the use of rigorous methods further contributes to restricting the estimates to only those supported by strong evidence and to ICD disease categories rather than including all health outcomes. Development of additional estimates would contribute to obtaining a fuller picture of the population health impacts from chemicals.

This review is also limited in its scope: Not all chemicals have been reviewed here, but only toxic exposures to chemicals which can be significantly reduced or eliminated through environmental and occupational management as described in the background section. Therefore lifestyle issues, such as active smoking or other substance abuse, have not been taken into account here. The same applies to chemicals acting on health through radiation rather than their toxic properties.

Significant examples of chemicals with yet unknown burden of disease include: a) chronic exposure to toxic pesticides; b) exposure to mercury c) exposure to cadmium, d) exposure to additional occupational carcinogens. It is, unfortunately, not possible to conduct estimates based on the different modes of action by which chemicals exert their toxic effects, such as through endocrine, immune or other systems.

**Figure 3:**
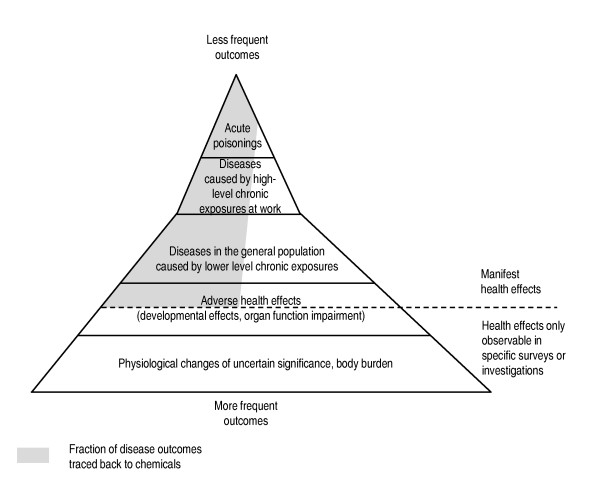
**Occurrence and detection of health impacts from chemicals Adapted from **[64,65]

While certain outcomes, such as those resulting from acute poisonings or high-level exposures, may easily be traced back to chemicals, other delayed or sub-clinical health effects such as cancers or certain neurological diseases are much more difficult to allocate to specific exposures (Figure 3). This is particularly true for diseases with long lag-times from exposures, complex exposure assessment, and often non-specific health outcomes. Also current toxicological test systems have limitations in their ability to predict effects in humans. Figure 2 schematically represents the possible fraction of the true burden of disease from chemical exposure that has effectively been traced back with sufficient scientific evidence to chemicals. It highlights that acute poisonings, outcomes caused by high-level exposures and rare health effects in more controlled occupational environments are often easier to trace back to chemicals than health effects resulting from the more frequent but lower-level exposures.

Uncertainty estimates were rarely available for the identified burden of disease estimates, and even if they were, they mostly relied on expert opinion or variation of selected input parameters rather than more systematic assessments. This was due to difficulties in the evaluation of the numerous uncertainties inherent to burden of disease estimates, which stem from exposure and exposure-response relationships, their extrapolation from one population to another or other parameter or model uncertainties. Sources of uncertainties were different for each of the assessed risk factors, and additional information on their specific sources can be found in the original publications (see references in Table 3).

The estimated disease burden does not show the important beneficial effects of long-term regulation of chemicals in food, consumer and other chemical products, industrial emissions and workers' protection which have already prevented a significant fraction of the disease burden that would have occurred had these controls not been in place. This is due to the fact that the burden of disease estimates represent a "snapshot" for about the year 2004, and limited information on trends. For example, in 2003, the European Commission forecast the health benefits of its REACH legislation to be 50 billion Euros over 30 years [60].

To improve exposure information, bio-monitoring initiatives are developing, and should continue improving large-scale exposure information [61,62]. New strategies and initiatives have been implemented which might change the picture of chemical exposure and risk assessment and could improve estimation of disease burden caused by chemicals (e.g. the REACH programme [60], the High Throughput Screening Initiative [63]). In addition, alternative, innovative methods may be required to estimate the full health impacts involving chemicals at population level, using for example a combination of disease trends, exposure patterns, biomarkers, and an improved understanding of the human relevance of effects seen in chemical testing systems.

### Link to policy initiatives

In 2002 at the World Summit on Sustainable Development (WSSD), governments renewed their commitment to the sound management of chemicals throughout their life cycle and of hazardous wastes. Governments aim to assure, by 2020, that chemicals are used and produced in ways that lead to the minimization of significant adverse effects on human health and the environment, using transparent science-based risk assessment procedures and science-based risk management procedures. The burden of disease information presented here shows that this goal has not been reached, and the limited available trend information indicates that renewed efforts will be required over the next decade to 2020. This is despite the knowledge that has long existed about the adverse health impacts of lead, mercury, asbestos and the other chemicals considered in this paper.

While the estimation of the burden of disease attributable to air pollution and naturally occurring chemicals has involved exposure and risks assessments by health or environment authorities, managing these exposures and reducing risk often requires action by other sectors and stakeholders, including regulatory bodies, industry and civil society. These actors are often different than those responsible for the sound management of agricultural and industrial chemicals. For example, health impacts and exposure to air pollution can primarily be modified through action in the energy and transport sectors and the industry, while arsenic in drinking-water is managed through the water sector. Other exposures involve chemicals only as a side-product of energy generation or of tobacco consumption, such as the main contributions to outdoor and indoor air pollution. Reducing human exposure to air pollutants and naturally occurring chemicals therefore requires efforts from a wide range of stakeholders, including industry and civil society.

Our review indicates that public health can be improved substantially, and in some cases relatively quickly, by identifying and implementing further effective interventions on chemicals of major public health concern including those addressed in this article. It is particularly urgent to address those chemical such lead and asbestos for which evidence, exposure and policy options have been known for quite some time, international agreements are in place, but exposure and effects are still significant in several populations. In addition governments can improve public health by collecting information to identify the additional hazardous chemicals to which their populations are exposed in order to take action on the most important problems. Finally, research aiming to improve our understanding of the link between chemicals and negative health impacts is imperative in order to prioritize actions and assess their effectiveness.

## Conclusions

This review shows that the currently known disease burden from chemicals is large, and that the yet unknown burden may be considerable. Although underestimated, the global burden of disease attributable to chemicals is useful information for international, regional and national decision-makers from the different sectors and programmes who have a role to play in reducing human exposure to toxic chemicals (Figure 1). This review supports that further attention should focus on investigating population health impacts from chemicals, and on the preventive measures limiting harmful exposures to chemicals.

The estimated burden of disease from chemicals and its preventable fraction provides an indication of how much disease burden could be prevented through targeted action, and will facilitate the evaluation and monitoring of these actions in the future. For example the proportion of people with blood lead levels above 10 ug/dl globally decreased from 20% to 14%, alongside similar reductions in the disease burden it caused. This reduction is due mostly to the phase out of leaded gasoline in most countries, providing a powerful example of the impact risk management can have in a relatively short time.

## Abbreviations

COPD: Chronic Obstructive Pulmonary Disease; CRA: Comparative Risk Assessment; DALY: Disability Adjusted Life Years; ICD: International Classification of Diseases; IQ: Intellectual Quotient; IARC: International Agency for Research on Cancer; PAH: Polycyclic Aromatic Hydrocarbon; REACH: Registration, Evaluation, Authorisation and Restriction of Chemicals; WHO: World Health Organization.

## Competing interests

The authors declare that they have no competing interests.

## Authors' contributions

APU reviewed the literature, synthesized the data and drafted the main part of the manuscript. PH provided input to the drafting process. CV drafted the section 'link to policy initiatives' and reviewed the draft. Roberto Bertollini provided overall guidance and reviewed the draft. All authors read and approved the final manuscript.

## Supplementary Material

Additional file 1 **Search terms used in Pubmed**. Contains search terms used in the search of articles in Pubmed databases Click here for file

## References

[B1] International Programme on Chemical Safety Human exposure assessment Environmental Health Criteria, No. 214 2000 Geneva: World Health Organization http://www.inchem.org/documents/ehc/ehc/ehc214.htm

[B2] International Programme on Chemical Safety Principles for evaluating health risks in children associated with exposure to chemicals Environmental Health Criteria, No. 237 2008 Geneva: World Health Organization http://www.inchem.org/documents/ehc/ehc/ehc237.pdf

[B3] World Health Organization Air Quality Guidelines-Global Update 2005. Particulate matter, ozone, nitrogen dioxide and sulfur dioxide 2005 Copenhagen: World Health Organization Regional Office for Europe http://whqlibdoc.who.int/hq/2006/WHO_SDE_PHE_OEH_06.02_eng.pdf

[B4] World Health Organization Guidelines for drinking-water quality 2008 3 Geneva http://www.who.int/water_sanitation_health/dwq/gdwq3rev/en/

[B5] Levin ML The occurrence of lung cancer in man Acta Unio Int Contra Cancrum 1953 9 531 541 13124110

[B6] Miettinen OS Proportion of disease caused or prevented by a given exposure, trait or intervention Am J Epidemiol 1974 99 325 332 4825599 10.1093/oxfordjournals.aje.a121617

[B7] Murray C Lopez AD The global burden of disease 1996 Geneva: World Health Organization, Harvard School of Public Health, World Bank

[B8] Ezzati M Lopez AD Rodgers A Vander Hoorn S Murray CJL Selected major risk factors and global and regional burden of disease Lancet 2002 360 1347 1360 10.1016/S0140-6736(02)11403-6 12423980

[B9] Ezzati M Lopez AD Rodgers A Murray CJL (Eds) Comparative Quantification of Health Risks 2004 Geneva: World Health Organization http://www.who.int/publications/cra/en/

[B10] World Health Organization The World Health Report 2002 - Reducing risks, promoting healthy life 2002 Geneva http://www.who.int/whr/2002/en/index.html 10.1080/1357628031000116808 14741909

[B11] Lokuge KM Smith W Caldwell B Dear K Milton AH The effect of arsenic mitigation interventions on disease burden in Bangladesh Environ Health Perspect 2004 112 1172 1177 10.1289/ehp.6866 15289162 PMC1247477

[B12] Gunnell D Eddleston M Phillips MR Konradsen F The global distribution of fatal pesticide self-poisoning: systematic review BMC Public Health 2007 7 357 10.1186/1471-2458-7-357 18154668 PMC2262093

[B13] Prüss-Ustün A Corvalan C Preventing disease through healthy environments: Towards an estimate of the environmental burden of disease 2006 Geneva: World Health Organization http://www.who.int/quantifying_ehimpacts/publications/preventingdisease/en/index.html

[B14] World Health Organization Global health risks: mortality and burden of diseases attributable to selected major risks 2009 Geneva http://www.who.int/healthinfo/global_burden_disease/global_health_risks/en/index.html

[B15] Concha-Barrientos M Imel Nelson D Driscoll T Steenland NK Punnett L Fingerhut MA Prüss-Üstün A Leigh J Tak SW Corvalàn C Ezzati M, Lopez AD, Rodgers A, Murray CJL Selected occupational risk factors Comparative quantification of health risks 2004 World Health Organization

[B16] Krieger R Hayes' Handbook of Pesticide Toxicology 2010 3 Amsterdam: Academic Press

[B17] Vijayakumar L Satheesh-Babu B Does 'no pesticide' reduce suicides? Int J Soc Psychiatry 2009 55 401 406 10.1177/0020764008095340 19617276

[B18] Gunnell D Fernando R Hewagama M Priyangika WDD Konradsen F Eddleston M The impact of pesticide regulations on suicide in Sri Lanka Int J Epidemiol 2007 36 1235 1242 10.1093/ije/dym164 17726039 PMC3154644

[B19] World Health Organization Chrysotile Asbestos Environmental Health Criteria, No. 203 1998 Geneva: World Health Organization http://www.inchem.org/documents/ehc/ehc/ehc203.htm

[B20] Driscoll T Nelson DI Steenland K Leigh J Concha-Barrientos M Fingerhut M Prüss-Ustün A The global burden of non-malignant respiratory disease due to occupational airborne exposures Am J Ind Med 2005 48 432 45 10.1002/ajim.20210 16299701

[B21] Baan R Secretan B Bouvard V Benbrahim-Tallaa Guha N Freeman C Galichet L Cogliano V Special Report: Policy; A review of human carcinogens--Part F: Chemical agents and related occupations Lancet Oncology 2009 10 1443 1444 10.1016/S1470-2045(09)70358-4 19998521

[B22] Rom WN Markowitz SB Environmental and occupational medicine 2007 Philadelphia: Lippincott Williams & Wilkins

[B23] World Health Organization Health aspects of air pollution 2004 Copenhagen: World Health Organization Regional Office for Europe http://www.euro.who.int/document/E83080.pdf

[B24] Cohen AJ Anderson HR Ostro B Pandey KD Krzyzanowski M Künzli N Gutschmidt K Pope CA III Romieu I Samet JM Smith KR Ezzati M, Lopez AD, Rodgers A, Murray CJL Urban air pollution Comparative quantification of health risks 2004 World Health Organization

[B25] Sastry N Forest fires, air pollution, and mortality in southern Asia Demography 2002 39 1 23 10.1353/dem.2002.0009 11852832

[B26] Smith KR Mehta S Maeusezahl-Feuz M Ezzati M, Lopez AD, Rodgers A, Murray CJL Indoor air pollution from solid household fuels Comparative quantification of health risks 2004 World Health Organization

[B27] International Agency for Research on Cancer Tobacco smoke and involuntary smoking IARC Monograph on the Evaluation of Carcinogenic Risks to Humans, No. 83] 2004 Lyon: World Health Organization http://monographs.iarc.fr/ENG/Monographs/vol83/index.php PMC4781536 15285078

[B28] United States Surgeon General The Health Consequences of Involuntary Exposure to Tobacco Smoke: A Report of the Surgeon General 2006 Atlanta: United States Department of Health and Human Services, Centers for Disease Control and Prevention, Coordinating Center for Health Promotion, National Center for Chronic Disease Prevention and Health Promotion, Office on Smoking and Health http://www.surgeongeneral.gov/library/secondhandsmoke/

[B29] Öberg M Woodward A Jaakkola MS Peruga A Prüss-Ustün A Worldwide burden of disease from exposure to second-hand smoke: a retrospective analysis of data from 192 countries Lancet 2011 377 139 146 21112082 10.1016/S0140-6736(10)61388-8

[B30] Fewtrell L Kaufmann RB Prüss-Ustün A Lead: Assessing the environmental burden of disease at national and local levels 2003 Geneva: World Health Organization http://www.who.int/quantifying_ehimpacts/publications/9241546107/en/index.html

[B31] Prüss-Ustün A Fewtrell L Landrigan P Ayuso-Mateos JL Ezzati M, Lopez AD, Rodgers A, Murray CJL Lead exposure Comparative quantification of health risks 2004 World Health Organization

[B32] Meyer PA Brown MJ Falk H Global approach to reducing lead exposure and poisoning Mutat Res 2008 659 166 175 10.1016/j.mrrev.2008.03.003 18436472

[B33] Haefliger P Mathieu-Nolf M Lociciro S Ndiaye C Coly M Diouf A Lam Faye A Sow A Tempowski J Pronczuk J Filipe AP Bertollini R Neira M Mass Lead Intoxication from Informal Used Lead-Acid Battery Recycling in Dakar, Senegal Environ Health Perspect 2009 117 1535 1540 20019903 10.1289/ehp.0900696 PMC2790507

[B34] International Agency for Research on Cancer Arsenic in drinking-water IARC Monographs on the evaluation of carcinogenic risks to humans, no. 84 2004 Lyon: World Health Organization

[B35] World Health Organization Arsenic and Arsenic Compounds Environmental Health Criteria, no. 224 2001 World Health Organization

[B36] Smith AH Steinmaus CM Health effects of arsenic and chromium in drinking water: recent human findings Annu Rev Public Health 2009 30 107 122 10.1146/annurev.publhealth.031308.100143 19012537 PMC2762382

[B37] Fewtrell L Fuge R Kay D An estimation of the global burden of disease due to skin lesions caused by arsenic in drinking water J Water Health 2005 3 101 107 16075937

[B38] Committee on Fluoride in Drinking Water, National Research Council Fluoride in Drinking Water: A Scientific Review of EPA's Standards 2006 Washington: National Research Council of the National Academy http://www.nap.edu/catalog.php?record_id=11571

[B39] Fewtrell L Smith S Kay D Bartram J An attempt to estimate the global burden of disease due to fluoride in drinking water J Water Health 2006 4 533 542 17176823

[B40] Cohen JT Bellinger DC Shaywitz BA A quantitative analysis of prenatal methyl mercury exposure and cognitive development Am J Prev Med 2005 29 353 365 10.1016/j.amepre.2005.06.007 16242602

[B41] Poulin J Gibb H Mercury: Assessing the environmental burden of disease at national and local levels Environmental Burden of Disease Series, no. 16 2008 Geneva: World Health Organization

[B42] Cohen J Bellinger DC Shaywitz B A quantitative analysis of prenatal methyl mercury exposure and cognitive development Am J Prev Med 2005 29 353 65 10.1016/j.amepre.2005.06.007 16242602

[B43] Spadaro JV Rabl A Global Health Impacts and Costs Due to Mercury Emissions Risk Analysis 2008 28 603 613 10.1111/j.1539-6924.2008.01041.x 18643818

[B44] European Environment Agency AirBase http://www.eea.europa.eu/themes/air/airbase

[B45] World Bank The little green data book 2006 Washington http://siteresources.worldbank.org/INTEEI/936214-1146251511077/20916989/LGDB2006.pdf

[B46] World Health Organization WHO Household energy database http://www.who.int/indoorair/health_impacts/he_database/en/

[B47] Fewtrell LJ Prüss-Ustün A Landrigan P Ayuso-Mateos JL Estimating the global burden of disease of mild mental retardation and cardiovascular diseases from environmental lead exposure Environ Res 2004 94 120 133 10.1016/S0013-9351(03)00132-4 14757375

[B48] Finnish Institute of Occupational Health CAREX - International Information System on Occupational Exposure to Carcinogens http://www.ttl.fi/en/chemical_safety/carex/Pages/default.aspx

[B49] Navas-Acien A Guallar E Silbergeld EK Rothenberg SJ Lead exposure and cardiovascular disease - a systematic review Environ Health Perspect 2007 115 472 482 10.1289/ehp.9785 17431501 PMC1849948

[B50] Schwartz J Lead, blood pressure, and cardiovascular disease in men Arch Environ Health 1995 50 31 37 10.1080/00039896.1995.9955010 7717767

[B51] Lanphear BP Hornung R Khoury J Yolton K Baghurst P Bellinger DC Canfield RL Dietrich KN Bornschein R Greene T Rothernberg S Needleman H Schnaas L Wasserman G Graziano J Roberts R Low-level environmental lead exposure and children's intellectual function: an international pooled analysis 2005 113 Environ Health Perspect 894 899 10.1289/ehp.7688 PMC1257652 16002379

[B52] Schwartz J Low-level lead exposure and children's IQ: a meta-analysis and search for a threshold Environ Res 1994 65 42 55 10.1006/enrs.1994.1020 8162884

[B53] Pope CA Burnett RT Thun MJ Calle EE Krewski D Ito K Thurston GD Lung cancer, cardiopulmonary mortality, and long-term exposure to fine particulate air pollution JAMA 2002 287 1132 1141 10.1001/jama.287.9.1132 11879110 PMC4037163

[B54] California Environmental Protection Agency Proposed Identification of Environmental Tobacco Smoke as a Toxic Air Contaminant 2005 Sacramento: California Environmental Protection Agency, Air Resources Board http://oehha.ca.gov/air/environmental_tobacco/2005etsfinal.html

[B55] Tsai SM Wang TN Ko YC Mortality for certain diseases in areas with high levels of arsenic in drinking water Arch Environ Health 1999 54 186 193 10.1080/00039899909602258 10444040

[B56] Steenland K Burnett C Lalich N Ward E Hurrell J Dying for work: The magnitude of US mortality from selected causes of death associated with occupation Am J Ind Med 2003 43 461 482 10.1002/ajim.10216 12704620

[B57] Steenland K Loomis D Shy C Simonsen N Review of occupational lung carcinogens Am J Ind Med 1996 29 474 490 10.1002/(SICI)1097-0274(199605)29:5<474::AID-AJIM6>3.0.CO;2-M 8732921

[B58] World Health Organization The global burden of disease: 2004 update 2009 Geneva http://www.who.int/healthinfo/global_burden_disease/2004_report_update/en/index.html

[B59] McCartor A Becker D Blacksmith Institute's world's worst pollution problems report 2010 2010 New York: Blacksmith Institute http://www.worstpolluted.org/2010-report.html Accessed November 16, 2010

[B60] Commission of the European Communities Commission staff working paper "Regulation of the European Parliament and of the Council concerning the Registration, Evaluation, Authorisation and Restrictions of Chemicals (REACH), establishing a European Chemicals Agency and amending Directive 1999/45/EC and Regulation (EC) on Persistent Organic Pollutants "1 Extended impact assessment" 2003 Brussels COM(2003)644 final

[B61] Centers for Disease Control Fourth national report on human exposure to environmental chemicals. Updated tables, July 2010 2010 Atlanta http://www.cdc.gov/exposurereport/pdf/Update_Tables.pdf

[B62] Viso AC Casteleyn L Biot P Eilstein D Human biomonitoring programmes and activities in the European Union. Journal of Epidemiology and Community Health 2009 63 623 624 19596843 10.1136/jech.2008.083709

[B63] National Toxicology Programme High throughput screening initiative http://ntp.niehs.nih.gov/?objectid=05F80E15-F1F6-975E-77DDEDBDF3B941CD

[B64] de Hollander AEM Melse JM Lebret E Kramers PGN An aggregate public health indicator of the impact of multiple environmental exposures Methodology for assessment of environmental burden of disease 2000 Geneva: World Health Organization

[B65] de Hollander AE Melse JM Lebret E Kramers PG An aggregate public health indicator to represent the impact of multiple environmental exposures Epidemiology 1999 10 606 617 10.1097/00001648-199909000-00030 10468440

[B66] Klaassen C Casarett Doull's Toxicology The Basic Science of Poisons 2008 7 USA: McGraw Hill http://www.mcgraw-hill.co.uk/html/0071470514.html

[B67] LaDou J Current occupational & environmental medicine 2004 3 New York: McGraw-Hill

[B68] Boschetto P Quintavalle S Miotto D Lo Cascio N Zeni E Mapp CE Chronic obstructive pulmonary disease (COPD) and occupational exposures J Occup Med Toxicol 2006 1 11 10.1186/1745-6673-1-11 16756686 PMC1513231

[B69] Balmes J Becklake M Blanc P Henneberger P Kreiss K Mapp C Milton D Schwartz D Toren K Viegi G American Thoracic Society Statement: Occupational contribution to the burden of airway disease Am J Respir Crit Care Med 2003 167 787 797 10.1164/rccm.167.5.787 12598220

[B70] Jeebhay MF Quirce S Occupational asthma in the developing and industrialised world: a review Int J Tuberc Lung Dis 2007 11 122 133 17263280

[B71] Jaakkola JJK Jaakkola MS Professional cleaning and asthma Curr Opin Allergy Clin Immunol 2006 6 85 90 10.1097/01.all.0000216849.64828.55 16520670

[B72] Bardana EJ 10. Occupational asthma J Allergy Clin Immunol 2008 121 S408 411 quiz S421 10.1016/j.jaci.2007.08.005 18241692

[B73] Longnecker MP Klebanoff MA Zhou H Brock JW Association between maternal serum concentration of the DDT metabolite DDE and preterm and small-for-gestational-age babies at birth Lancet 2001 358 110 114 10.1016/S0140-6736(01)05329-6 11463412

[B74] Taha TE Gray RH Agricultural pesticide exposure and perinatal mortality in central Sudan Bull World Health Organ 1993 71 317 321 8324850 PMC2393503

[B75] Zhang J Cai WW Lee DJ Occupational hazards and pregnancy outcomes Am J Ind Med 1992 21 397 408 10.1002/ajim.4700210312 1585950

[B76] Whyatt RM Camann D Perera FP Rauh VA Tang D Kinney PL Garfinkel R Anrews H Hoepner L Barr DB Biomarkers in assessing residential insecticide exposures during pregnancy and effects on fetal growth Toxicol Appl Pharmacol 2005 206 246 254 10.1016/j.taap.2004.11.027 15967215

[B77] Nurminen T Maternal pesticide exposure and pregnancy outcome J Occup Environ Med 1995 37 935 940 10.1097/00043764-199508000-00008 8520956

[B78] Wigle DT Arbuckle TE Turner MC Bérubé A Yang Q Liu S Krewski D Epidemiologic evidence of relationships between reproductive and child health outcomes and environmental chemical contaminants J Toxicol Environ Health B Crit Rev 2008 11 373 517 18470797 10.1080/10937400801921320

[B79] Clapp RW Jacobs MM Loechler EL Environmental and occupational causes of cancer: new evidence 2005-2007 Rev Environ Health 2008 23 1 37 18557596 10.1515/reveh.2008.23.1.1 PMC2791455

[B80] Henry SH Bosch FX Bowers JC Aflatoxin, hepatitis and worldwide liver cancer risks Adv Exp Med Biol 2002 504 229 233 11922091 10.1007/978-1-4615-0629-4_24

[B81] Irigaray P Newby JA Clapp R Hardell L Howard V Montagnier L Epstein S Belpomme D Lifestyle-related factors and environmental agents causing cancer: an overview Biomed Pharmacother 2007 61 640 658 10.1016/j.biopha.2007.10.006 18055160

[B82] Wogan GN Hecht SS Felton JS Conney AH Loeb LA Environmental and chemical carcinogenesis Semin Cancer Biol 2004 14 473 486 10.1016/j.semcancer.2004.06.010 15489140

[B83] Axelrad DA Bellinger DC Ryan LM Woodruff TJ Dose-response relationship of prenatal mercury exposure and IQ: an integrative analysis of epidemiologic data Environ Health Perspect 2007 115 609 615 10.1289/ehp.9303 17450232 PMC1852694

[B84] Grandjean P Landrigan PJ Developmental neurotoxicity of industrial chemicals Lancet 2006 368 2167 2178 10.1016/S0140-6736(06)69665-7 17174709

[B85] Bhatnagar A Environmental cardiology: studying mechanistic links between pollution and heart disease Circ Res 2006 99 692 705 10.1161/01.RES.0000243586.99701.cf 17008598

[B86] Bertazzi PA Consonni D Bachetti S Rubagotti M Baccarelli A Zocchetti C Pesatori AC Health effects of dioxin exposure: a 20-year mortality study Am J Epidemiol 2001 153 1031 1044 10.1093/aje/153.11.1031 11390319

[B87] Longnecker MP Daniels JL Environmental contaminants as etiologic factors for diabetes Environ Health Perspect 2001 109 871 876 10.2307/3454649 11744505 PMC1240622

[B88] Remillard RBJ Bunce NJ Linking dioxins to diabetes: epidemiology and biologic plausibility Environ Health Perspect 2002 110 853 858 10.1289/ehp.02110853 12204817 PMC1240982

[B89] Tseng C Tseng C Chiou H Hsueh Y Chong C Chen C Epidemiologic evidence of diabetogenic effect of arsenic Toxicol Lett 2002 133 69 76 10.1016/S0378-4274(02)00085-1 12076511

[B90] World Health Organization Principles and methods for assessing autoimmunity associated with exposure to chemicals 2006 Geneva: World Health Organization http://www.inchem.org/documents/ehc/ehc/ehc236.pdf

[B91] Spiewak R Pesticides as a cause of occupational skin diseases in farmers Ann Agric Environ Med 2001 8 1 5 11426918

[B92] Presgrave RDF Camacho LAB Villas Boas MHS A profile of unintentional poisoning caused by household cleaning products, disinfectants and pesticides Cad Saude Publica 2008 24 2901 2908 10.1590/S0102-311X2008001200019 19082281

[B93] Klepac T Busljeta I Macan J Plavec D Turk R Household chemicals--common cause of unintentional poisoning Arh Hig Rada Toksikol 2000 51 401 407 11276967

[B94] Eddleston M Patterns and problems of deliberate self-poisoning in the developing world QJM 2000 93 715 731 10.1093/qjmed/93.11.715 11077028

[B95] Corbett JJ Winebrake JJ Green EH Kasibhatla P Eyring V Lauer A Mortality from ship emissions: a global assessment Environ Sci Technol 2007 41 8512 8518 10.1021/es071686z 18200887

