# Supplementary material for: Knowns and unknowns on burden of disease due to chemicals: a systematic review
Source: Environ Health. 2011 Jan 21;10:9. doi: 10.1186/1476-069X-10-9 (PMC3037292; doi:10.1186/1476-069X-10-9)
Supplement: Additional file 1 — Search terms used in Pubmed. Contains search terms used in the search of articles in Pubmed databases [file 1476-069X-10-9-S1.PDF]

Additional file 1: Search terms used in PubMed databases

The search combined the following terms:

- Global (in the title) or "world health" (as Mesh term),
- Humans (as Mesh term),

and any of the following MeSH terms:

Air pollutants or pollution
Aflatoxins
Arsenic
Asbestos
Benzene
Cadmium
Carbon monoxide
Carcinogens
Chromium
Diesel exhaust
DDT
Dioxins
Dust
Dyes
Environmental exposure
Flame retardants
Fluoride
Formaldehyde
Furans
Hazardous chemicals
Heavy metals
Hexachlorobenzene
Hydrocarbons
Lead poisoning
Mercury
Nickel
Nitrates
Ozone
Paints
Particulate matter
Pesticides
Poisoning
Polybrominated biphenyls
Polychlorinated biphenyls
Radioactive pollutants
Selenium
Solvents
Smoke
Soil pollutants
Tobacco smoke pollution
Toluene
Vehicle emissions
Volatile organic compounds
Water pollutants
